# Reduction of the Measurement Time by the Prediction of the Steady-State Response for Quartz Crystal Microbalance Gas Sensors

**DOI:** 10.3390/s18082475

**Published:** 2018-07-31

**Authors:** Diana L. Osorio-Arrieta, José L. Muñoz-Mata, Georgina Beltrán-Pérez, Juan Castillo-Mixcóatl, Claudia O. Mendoza-Barrera, Víctor Altuzar-Aguilar, Severino Muñoz-Aguirre

**Affiliations:** 1 Facultad de Ciencias de Físico-Matemáticas, Benemérita Universidad Autónoma de Puebla, Av. San Claudio y Rio Verde, Col. San Manuel, CU., C.P. 72570 Puebla, Mexico; dloa_azul19@yahoo.com.mx (D.L.O.-A.); gbeltran@fcfm.buap.mx (G.B.-P.); jcastill@fcfm.buap.mx (J.C.-M.); cmendoza@fcfm.buap.mx (C.O.M.-B.); valtuzar@fcfm.buap.mx (V.A.-A.); 2 División de Mecatrónica, Universidad Tecnológica de Puebla, Antiguo Camino a la Resurrección 1002-A, Zona Industrial Oriente, C.P. 72300 Puebla, Mexico; jose.munoz@utpuebla.edu.mx

**Keywords:** QCM gas sensor, transient response, measurement time reduction, steady-state response prediction

## Abstract

This paper presents a new approach to reduce the measurement time by the prediction of the steady-state using the transient response to ethanol for quartz crystal microbalance gas sensors coated with ethyl cellulose. The experimentally measured response curves were successively fitted using a mathematical model based on the sum of two exponentials with different time constants. The parameters of the model were determined, and the time constants and the magnitude of the steady-state response were analyzed. Even though the time constants did not stabilize well, the parameter corresponding to the magnitude of the steady-state response quickly converged and stabilized after 37 s. Moreover, this calculated parameter was highly correlated with the measured values of the steady-state response, which was measured at five times the longest time constant (83 s) of the model. Therefore, the steady-state response could be predicted with a 55% reduction in the measurement (detection) time.

## 1. Introduction

The reduction of the measurement time of gas sensors is essential for the detection of gases that can be very dangerous for the human health, such as the inhalation of inert gases or any other poisonous gas which can provoke asphyxia and death. According to the European Industrial Gases Association (EIGA) [1] and DiMaio et al. [2], at low levels of oxygen, fainting can occur in 40 s and death within a few minutes. Some gases such as arsine and ammonia, among others, with a determined concentration can be lethal in less than 60 s, and many sensors perform the detection in 80 or 100 s [3,4,5], such that to decrease the detection time from 80 s to 40 s can be crucial to save lives. Measurement time reduction is essential primarily for sensors that have a slow response time, which is the case of the detection of substances in gas phase [6,7]. Metal-oxide semiconductor sensors are widely used and many of them present quite slow responses [8,9]. Moreover, for gas sensors based on quartz crystal microbalance (QCM) the response can be obtained in tens of seconds or even in minutes [10,11]. Therefore, reducing the measurement time of gas sensor response is currently very important since this allows the development of faster and more efficient systems for detection, recognition and classification of gases.

Although there are a variety of gas sensors, quartz crystal microbalance (QCM) gas sensors arrays have been frequently used in electronic noses development owing to their high correlation with the human nose and their simplicity of fabrication. Electronic noses use gas sensor arrays to imitate the human nose in detecting, recognizing, and discriminating odours. In fact, QCM sensors have been used for applications such as beverage or food quality control, cosmetology, and environment quality monitoring [12,13,14]. To recognize and discriminate odours and vapours from the response patterns of sensor arrays, both multivariate analysis methods and neural networks [15,16] are commonly used. Moreover, the steady-state response is the most used parameter for discriminating, classifying, and identifying a variety of compounds [17,18,19]. It is important to mention that the steady-state response of the gas sensors is directly related to the gas concentration in the environment. Although this method is reliable and effective, long measurement times are required to reach stabilization of the measured sensor response for optimal classification. Electronic noses are the most effective when they can be used to quickly discriminate different odours, especially in applications such as environmental quality monitoring or biomedical testing. To improve the quality of electronic noses, the measurement time needs to be reduced.

There are two main strategies to reduce the measurement time of gas sensors, the reduction by the response signal processing or the reduction of the sensor response time by changing the sensing film structure. Recently, some works have reported efforts in both directions, on one hand, the reduction of the measurement time by the transient response processing [20,21,22]. On the other hand, some authors are working on the sensor structure, for instance the reduction of the sensing film thickness [23,24] or introducing nanostructures inside the sensing film, such as nanocrystals or nanoclays [11,25], among others. However, reduction of the sensing film thickness means a decreasing of the sensor sensitivity and changing the sensing film structure means a further technological process that can be quite expensive. Therefore, to reduce the measurement time, we propose a new approach by the use of the transient response analysis based on successive fittings of the response curves using the Gauss-Newton method in order to predict the steady-state response. The method is quite simple, converges in very few iterations and the calculations can be performed very quickly in an algorithm implemented in a personal computer. This method has not been reported up to date.

In this study, we used QCM sensors coated with an ethyl-cellulose sensing film for detecting ethanol, a volatile organic compound frequently found in beverages or flavours and that is suitable to perform these kind of studies owing to its low toxicity. The sum of two exponential terms with different time constants was proposed as a model for studying the sensor curve. The sensor responses were obtained using our previously developed system with a resolution of 1 Hz [26]. The bi-exponential-model-based analysis showed that the steady-state response could be predicted in approximately 45% of the total response time.

## 2. QCM Sensor Operation Principle

**Figure 1:**
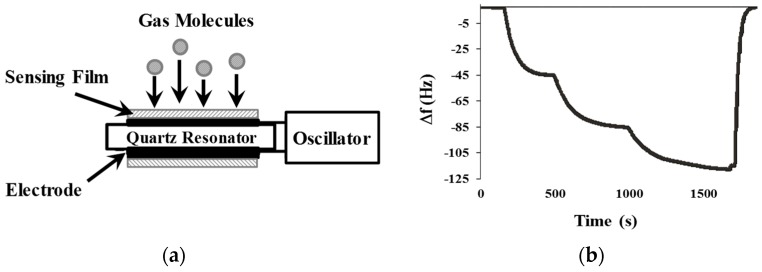
Operation principle of a QCM gas sensor. (**a**) Gas sensor mechanism. (**b**) Sensor response.

The principle of a QCM gas sensor operation is based on the interaction of gas molecules with the sensing film deposited over the gas sensor electrodes. When the mass inside the sensing film increases, the resonance frequency decreases owing to the mass-loading effect (Figure 1).

The Sauerbrey equation defines the behaviour of this frequency shift as a function of the mass change of the sensing film, the area of the crystal electrode, and the fundamental resonance frequency of the crystal [27], see Equation (1):

(1) $$\Delta f=-2.3\times 10^{-6}\cdot f_{0}^{2}\frac{\Delta m}{A}$$

where Δ*f* is the frequency shift (Hz), −2.3 × 10^−6^ is a constant obtained from the quartz density *ρ_q_* and its shear modulus *μ_q_*, Δ*m* is the mass (g) of the adsorbed gas molecules, *A* is the coated area (cm^2^) and *f*_0_ is the fundamental resonance frequency (Hz) of the crystal.

Moreover, coating the QCM with different kinds of sensing films allows fabrication of sensors with different characteristics. These sensors can be used to design sensor arrays to achieve more accurate measurements for gases or vapours.

## 3. Transient Response Analysis Model

In particular, the transient response can be useful for obtaining information on the interaction between the sensing film and the gas molecules, for discriminating odours and vapours, and for predicting the steady-state response [14]. Several studies have been performed using a model of the transient response of QCM sensors, such as odour classification [28,29], studies of different sensing films in order to separate and classify odour samples [30], and a direct study of the transient response curve using multi-exponential models [31,32]. However, to the best of our knowledge, there are no studies on the use of a transient response model to predict the steady-state response of QCM gas sensors exposed to volatile organic compounds, with the purpose of reducing the measurement time.

To observe the evolution of the transient response of QCM sensors, a high-resolution measurement instrument is required to obtain reliable data. In a previous work, we developed a system for measuring the response of an array of QCM gas sensors at a resolution of 1 Hz using a field-programmable gate array (FPGA) [26]. This system is suitable and it was used for performing transient response analysis.

**Figure 2:**
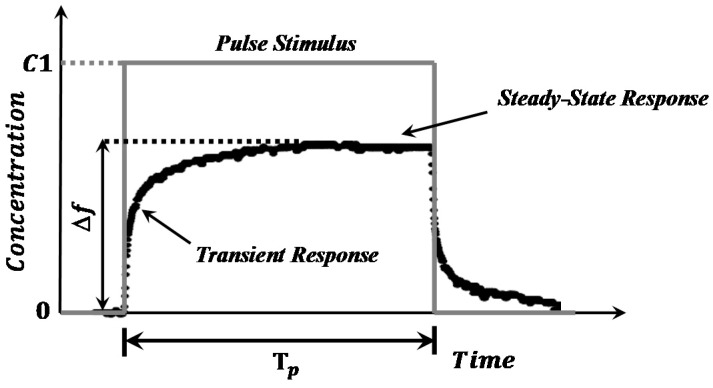
Dynamic response of the QCM gas sensor to a gas pulse stimulus.

A mathematical model of the sensor response is required to perform a transient response analysis. The sensor response can be studied using the rise and recovery regions; however, in this case, we are focusing on only the rise curve of the response in order to reduce the measurement time by predicting the steady-state response. Two assumptions were considered for this analysis. First, the input gas is supplied by a pulse function with an amplitude *C*1 and a time width T*_p_*, which represents a variation in the gas concentration applied to the sensor, as shown in Figure 2.

Second, the response curve can be represented by a second-order equation, namely, a sum of two exponential functions with different time constants [33]. Therefore, the bi-exponential model of the response is represented by:

(2) $$\Delta f\left(t\right)=c-\left[k_{1}\cdot exp\left(-\frac{t}{\tau_{1}}\right)+k_{2}\cdot exp\left(-\frac{t}{\tau_{2}}\right)\right]$$

where Δ*f* is the frequency shift of the QCM sensor, i.e., the sensor response, *c* is the parameter that represents the magnitude of the steady-state response at an infinite time (or at least after five times the longest time constant), *k*_1_ and *k*_2_ are constants that indicate the maximum amplitude of the exponentials, and *τ*_1_ and *τ*_2_ are the time constants associated with the two exponentials (note that *k*_1_ + *k*_2_ = *c*). The measured data of the sensor response obtained from a frequency counter were processed using a fitting process with the proposed model. The parameters of Equation (2) were obtained after convergence. The time constants were selected, and their evolution was observed as a function of time to analyze their influence in the prediction of the steady-state response.

## 4. Experimental Setup

### 4.1. Measurement System

The sensing film was deposited on the QCM electrodes using the casting method. The material solution was prepared inside a vial using a concentration of 0.5 mg/mL of ethyl cellulose in chloroform. A 2.5-μL droplet of the solution was placed on the surface of each electrode of a 12-MHz (AT-cut silver electrode) QCM crystal with a micropipette. The resulting frequency shift was Δ*f* = 25 KHz owing to deposition of the sensing film, which indicated an estimated film thickness of 0.66 μm.

**Figure 3:**
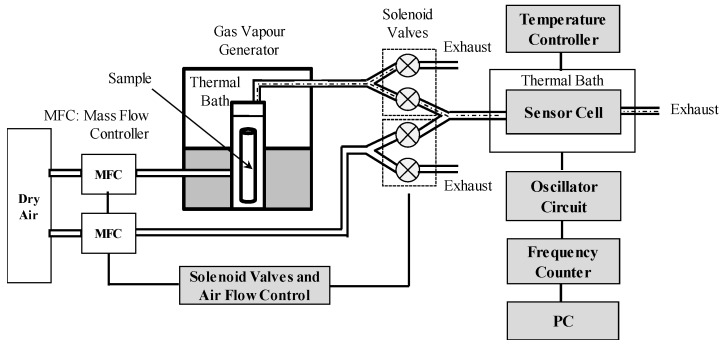
Experimental setup to measure the dynamic sensor response.

Figure 3 shows the setup for the sensor response measurement. The experimental measurement system used a homemade dynamic system to supply the gas/odour concentration. Inside the gas vapour generator, the liquid odour sample was contained in a vial and held at a constant temperature to allow sample evaporation. The odour sample used for the measurements was ethanol in different concentrations. The system supplied a constant concentration of the sample vapour diluted with a carrier gas, in this case dry-air was used to eliminate the humidity effects on the sensor response. The gas/odour pulse function was generated by two solenoid valves that were alternately activated by a microcontroller (PIC16F877, Microchip, Chandler, AZ, USA). The gas/odour sample travelled through a pipe toward the measurement cell. The QCM sensor was placed inside the measurement cell, which in turn was placed inside a thermal bath (RTE-10, Neslab, Newington, NH, USA) at a constant temperature of 20 °C. In order to achieve a correct measurement of the response, we must apply a gas stimulus very close to an ideal pulse. Otherwise we could be measuring the transient response of the system instead of that of the sensor. This condition was satisfied performing the measurements at a gas flow above 200 mL/min.

A homemade frequency counter was used to measure the frequency shift of the sensor. The frequency counter was designed using an FPGA and has a resolution of 1 Hz. The data were visualized, plotted, and stored using a virtual instrument in a personal computer [26].

### 4.2. Curve-Fitting Algorithm

An algorithm based on the Gauss–Newton method [34] was used to perform the fitting of the sensor response curve with the proposed bi-exponential model (Equation (2)). The model parameters to estimate were (*c*, *k*_1_, *k*_2_, *τ*_1_, *τ*_2_). The equation to calculate the new parameters based on the actual ones is expressed as:

(3) $$\theta_{n+1}=\theta_{n}+B_{n}$$

where *θ_n_* is the vector of the parameters (*c*, *k*_1_, *k*_2_, *τ*_1_, *τ*_2_). The initial parameters *θ*_0_ were defined by the program user. *B_n_* is a minimum estimator defined by:

(4) $$B_{n}={\left(J^{T}J\right)}^{-1}J^{T}\left(y\left(t\right)-\Delta f_{n}\right)\cdot B_{n}$$

where *y*(*t*) are the experimental data, Δ*f_n_* is the evaluated function using the current parameters and *J* is the Jacobian matrix defined by:

(5) $$J=\left(\frac{\partial \Delta f}{\partial c}\;\frac{\partial \Delta f}{\partial k_{1}}\;\frac{\partial \Delta f}{\partial \tau_{1}}\;\frac{\partial \Delta f}{\partial k_{2}}\;\frac{\partial \Delta f}{\partial \tau_{2}}\right)$$

**Figure 4:**
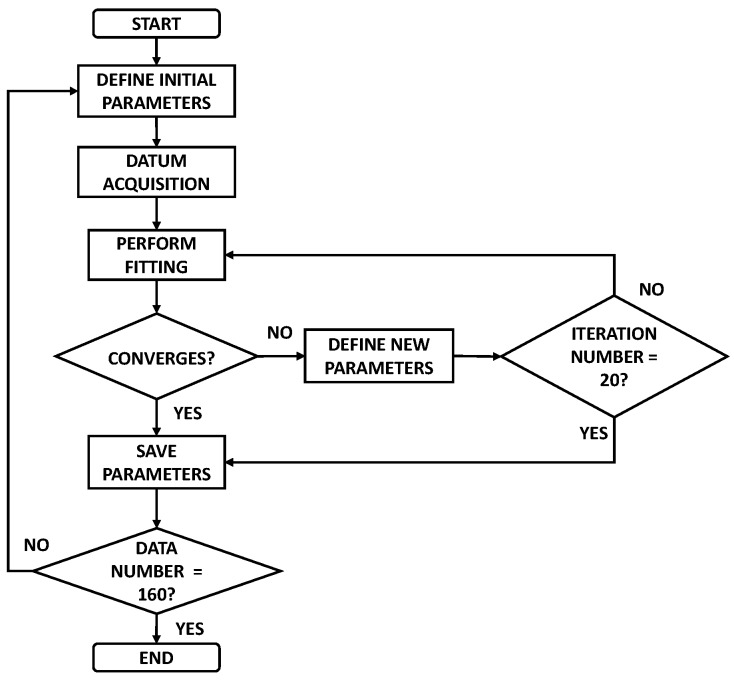
Flow chart of the curve fitting algorithm.

Figure 4 shows the flow chart for the algorithm implemented to perform the model calculation. The response curve was fitted each time that a measured datum was acquired. That is, the first datum of the sensor response was acquired, then the first curve fitting was performed, and the calculated parameters were stored. Then, the next datum was acquired, a new fitting was performed using the two data points, and the new parameters were calculated and stored. The fitting process did not converge using only a few data points, since the model parameters can take any value. For instance, for two data points, the *c* parameter can take a value as large as 1000 or as small as 0 and the same happens with the other parameters. It was clear that these parameters did not define the adequate fitting and the algorithm would continue working indefinitely. Therefore, if convergence was not reached, the fitting process was stopped at 20 iterations, since for our case if convergence was reached it occurred at 5 or 6 iterations at most. Even if the convergence was not reached the obtained model parameters were also stored for further analysis, as can be observed in Figure 4. 

Then, a new cycle was started by the definition of the initial parameters and a new datum was acquired, as is shown in the flow chart. As it was mentioned before, convergence of the fitting process for each acquired datum was reached as the amount of data increased above 37 s (37 data points). In this study, the process was stopped at a time of 160 s because, in all the cases it was a sufficient time since in the experimental measurements the steady-state was reached at a time below 100 s. The calculated model parameters were stored for further analysis, in particular the *c* parameter and, the time constants *τ*_1_ and *τ*_2_. It is important to mention that the initial parameters were the same for all the curve fittings performed. The execution time of the algorithm implemented is in the range of 3 ms to 100 ms for each arriving data point, which indicates that the fitting algorithm can be performed in real time since each datum was acquired each second.

## 5. Experimental Results and Discussion

A dynamic system was used to generate the input gas concentration in the form of a step function, which was applied to the gas sensors based on a QCM. Equation (2) was used to analyze the response. Several measurements were performed to collect sufficient data for the Gauss–Newton curve-fitting analysis and to determine suitable parameters for predicting the steady-state response using the data obtained from the frequency counter.

### 5.1. Curve-Fitting Algorithm Results

**Figure 5:**
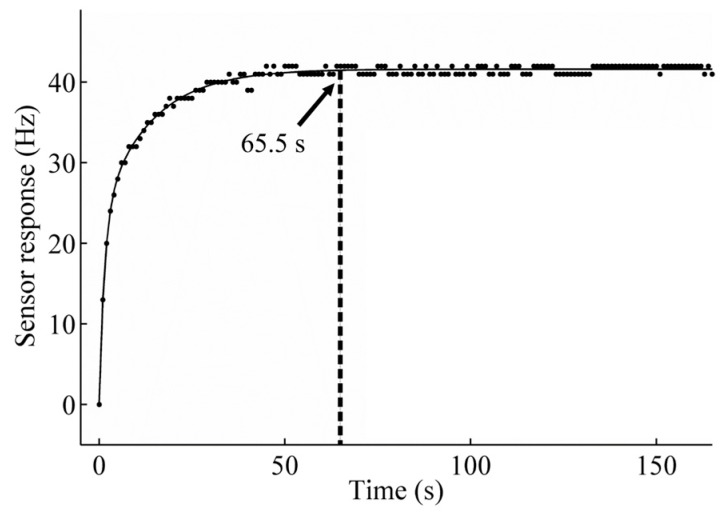
Curve fitting of the gas sensor response to ethanol.

As described in Section 4.2, the fitting parameters of the model in Equation (2) were calculated using the acquired data points until the steady-state response was well-established. Figure 5 shows an example of the fitting for the data obtained for a sample with an ethanol concentration of 1740 ppm. In this case, the response is plotted for a period of 160 s, and the continuous line shows the curve fitting performed. The fitting line clearly passed through several experimental points, and the correlation coefficient of R^2^ = 0.9895 confirmed a very good fitting of the model to the measured sensor response. The parameters calculated for this particular curve fitting were *c* = 41.5, *k*_1_ = 22.7, *k*_2_ = 18.8, *τ*_1_ = 1.4, and *τ*_2_ = 13.1.

We believe that the calculated time constants can be associated with two different phenomena. In particular, *τ*_1_ (the short time constant) can be related with the adsorption effect of gas molecules onto the sensing film surface because it is a relatively fast phenomenon. On the other hand, *τ*_2_ (the long time constant) can be related to the diffusion of gas molecules into the sensing film because it is a quite slow phenomenon as it was established in a previous work [33]. Therefore, since *τ*_2_ >> *τ*_1_, *τ*_2_ determines the time when the steady-state response is reached. By convention we consider that it occurred at 5*τ*_2_, which corresponds to 65.5 s in this particular case (see Figure 5).

### 5.2. Prediction of the Steady-State Sensor Response Using the C Parameter

**Figure 6:**
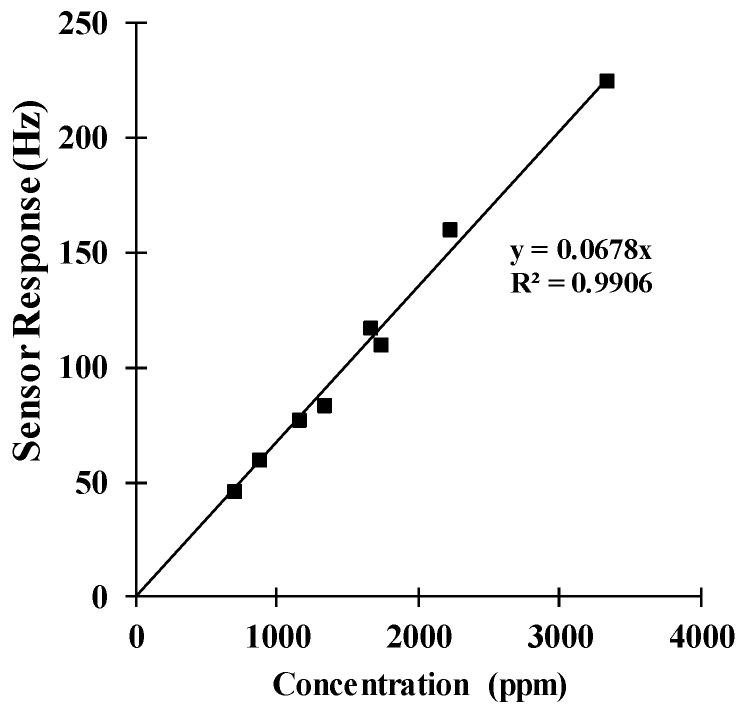
Sensor response from several measurements with respect to the ethanol concentration.

The prediction of the steady-state response magnitude of the QCM sensor from the experimental data can be used to optimize the measurement time (time of detection). Various measurements using different concentrations were performed to observe the sensor response behaviour, and the calculated time of 5*τ*_2_ was used to determine the measured steady-state response for such concentration values. Figure 6 shows the change in the frequency (sensor response) as a function of the applied concentration, where each point is the average of five measurements. As expected, the behaviour of the curve starting at a zero response was practically linear and had a correlation coefficient of R^2^ = 0.9906. Such behaviour is typically observed for QCM sensors for this range of concentrations.

An analysis of the evolution of the model parameters in function of time is essential for predicting the steady-state response of the sensor. Of particular interest are *τ*_1_, *τ*_2_ and the *c* parameter, which corresponds to the magnitude of the steady-state response.

**Figure 7:**
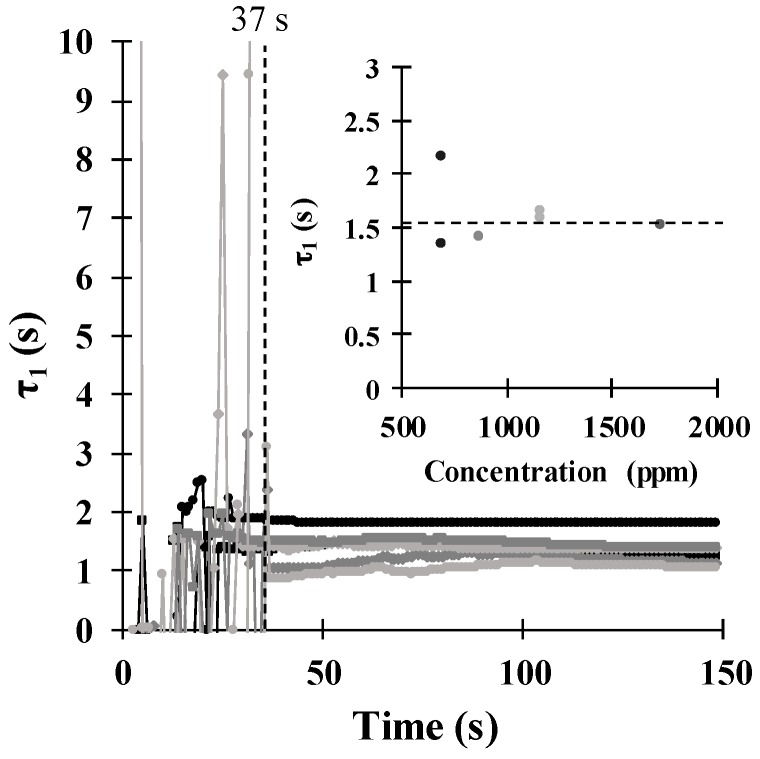
Evolution of the time constant *τ*_1_ in function of time, as calculated from experimental data. Inset: behaviour of *τ*_1_ as a function of the ethanol concentration.

The evolution in time of the first time constant τ_1_ is shown in Figure 7. In the first 36 s, *τ*_1_ showed highly fluctuating behaviour since the first fittings were performed with too few data points to reach convergence. In this case, the parameter can take any value. However, after 37 s, the parameter stabilized around a particular value with small fluctuations for different concentrations. In some measurements, this parameter stabilized at a shorter time of approximately 24 s. The value of *τ*_1_ had a very low magnitude and showed a constant dependency with the concentration, as illustrated in the inset of Figure 7. The average value was approximately 1.57 s with a dispersion of less than 9.04%. Therefore, *τ*_1_ was independent of the concentration, and we can say that this parameter was directly related to the superficial adsorption of ethanol molecules onto the sensing film. We thought that *τ*_1_ would stabilize in an earlier time of the response measurement since its value was too small (1.57 s), however, it still presents the influence of *τ*_2_, since both of them define the sensor response model.

**Figure 8:**
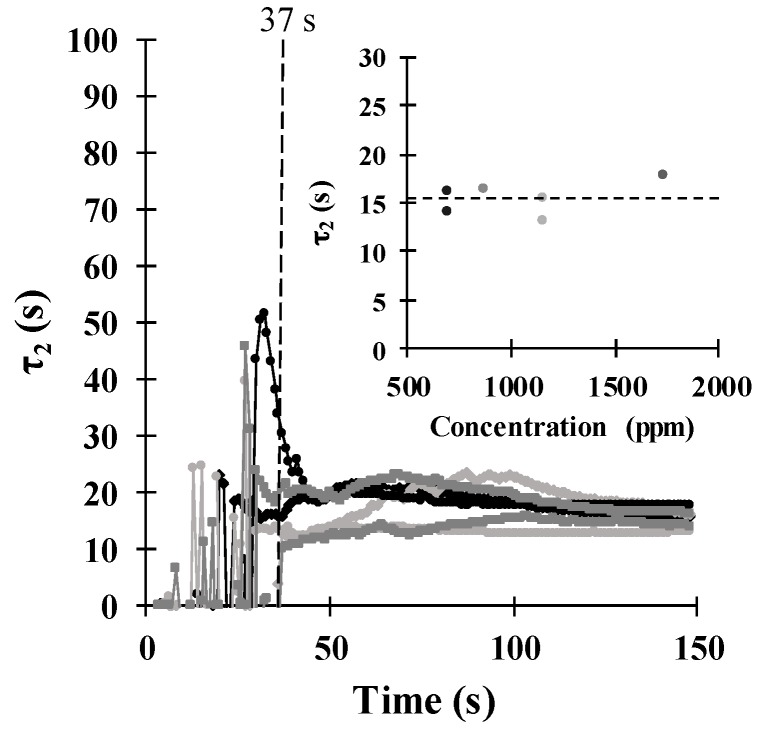
Evolution of the time constant *τ*_2_ with time, as calculated from experimental data. Inset: behaviour of *τ*_2_ as a function of the ethanol concentration.

Figure 8 shows the evolution of the *τ*_2_ parameter as a function of time. Similar to *τ*_1_, *τ*_2_ had highly fluctuating behaviour for the first 45 s, since the data points were not enough to reach stabilization. After this 45 s, the fluctuations decreased, and the parameter stabilized by approximately 130 s. Moreover, *τ*_2_ did not depend on the concentration, as shown in the inset of Figure 8, and maintained constant behaviour with a dispersion of 6.62%. The average value of this parameter was 15.6 s, which was much larger than that of *τ*_1_. Therefore, we assumed that this parameter can be associated with the diffusion of ethanol molecules into the sensing film because this process is a quite slow phenomenon. To corroborate this assumption, additional measurements should be performed in future work using sensors with different thicknesses or using different temperatures. The fact that this parameter did not stabilize in an earlier time of the response measurement can influence the stabilization of *τ*_1_ since both of them define the response model. However, even though *τ*_2_ did not stabilize soon, it can still be used as a criterion to define the time when the steady state was reached, which was taken as 5*τ*_2_ for practical cases.

**Figure 9:**
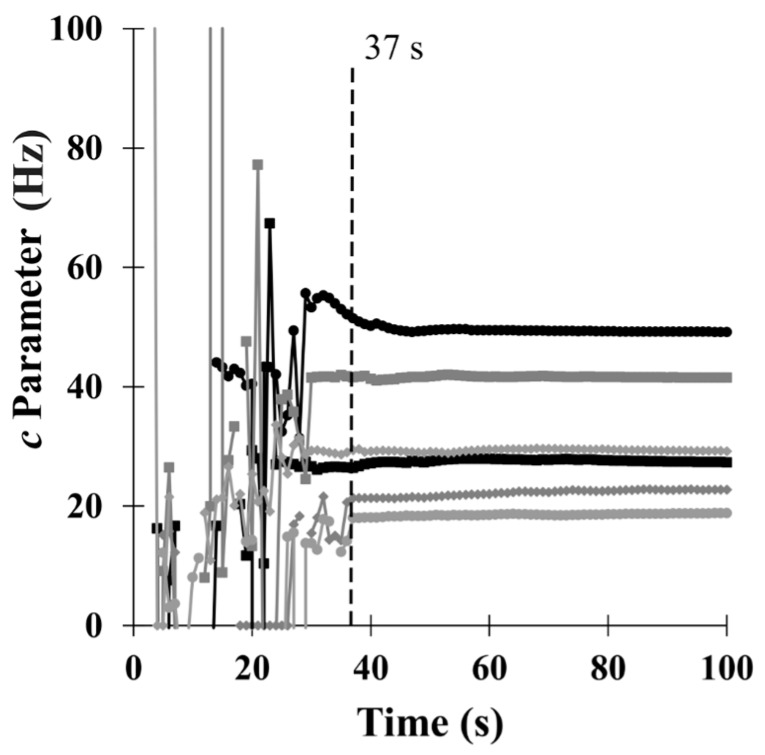
Evolution of the *c* parameter with time, as calculated from experimental data for different ethanol concentrations.

We then observed the evolution of the *c* parameter, which represents the magnitude of the steady-state response (see Equation (2)), as a function of time. The results of these calculations are shown for several concentrations in Figure 9. Similar to *τ*_1_ and *τ*_2_, the *c* parameter showed highly fluctuating behaviour until approximately 36 s due to the fact that the fitting process was performed using a small amount of data, convergence was not reached and the parameter can take any value. However, after 37 s, the parameter stabilized around a constant value with very small fluctuations. Even though *τ*_2_ did not achieved stabilization in 37 s, the *c* parameter did stabilize. Moreover, this stabilization occurred independently of the concentration. Therefore, we could obtain an estimate of the magnitude of the steady-state response without waiting until the steady-state was completely reached. Hence, this value was used as a prediction of the steady-state response.

**Figure 10:**
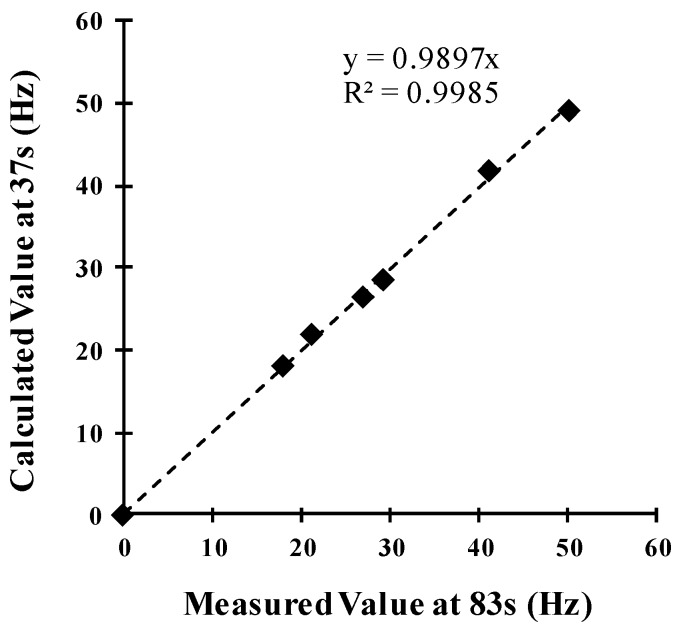
Comparison between the experimental steady-state response at 83 s and the calculated values of the *c* parameter at 37 s for different ethanol concentrations.

To corroborate the effectiveness of the prediction, we plotted the value calculated using the fitting model at 37 s with respect to the value experimentally measured at 83 s, which is equal to 5*τ*_2_, because it was assumed that the steady-state was reached at such time. The results shown in Figure 10 exhibit a linear relationship with a slope very close to 1.0 and a correlation coefficient of R^2^ = 0.9985. Therefore, the predicted value, which was calculated from the model in a time of 37 s, was equal to the value measured at a time of 83 s (5*τ*_2_). This result enabled a remarkable 55% reduction in the measurement time, which for poisonous or dangerous gases can be the difference between life and death, as it was mentioned above.

## 6. Conclusions

A new method for the measurement time reduction of sensor response was proposed using a bi-exponential model and a successive fitting of the sensor response curve to predict the steady state response. The study was performed for ethyl-cellulose coated QCM gas/odour sensors and the response to ethanol samples. The successive curve-fitting algorithm was implemented using the Gauss–Newton method to observe the evolution and behaviour of data obtained from several experimental measurements. The parameters of the mathematical model were calculated, in particular, the time constants (*τ*_1_, *τ*_2_) and the *c* parameter, which corresponds to the steady-state response. We found that the *c* parameter stabilized in 37 s, independently of the stabilization of the time constants. Moreover, the *c* parameter was highly correlated with the steady-state response of the sensor measured at a time of 5*τ*_2_ (five times the long time constant), which has a value of 83 s. Therefore, we showed that it is possible to predict the steady-state value from an analysis of the transient response, reducing the measurement (detection) time by 55%, which can be of vital importance in many cases, especially for poisonous or inert gases. Finally, the obtained time constants can be also used as discrimination parameters in sensor array systems at early stages of response measurement.
